# A multilevel mHealth drug abuse and STI/HIV preventive intervention for clinic settings in the United States: A feasibility and acceptability study

**DOI:** 10.1371/journal.pone.0221508

**Published:** 2019-08-22

**Authors:** David Cordova, Frania Mendoza Lua, Jaime Muñoz-Velázquez, Katie Street, Jose A. Bauermeister, Kathryn Fessler, Nicole Adelman, Youth Leadership Council, Torsten B. Neilands, Cherrie B. Boyer

**Affiliations:** 1 University of Michigan School of Social Work, Ann Arbor, Michigan, United States of America; 2 University of Chicago School of Social Service Administration, Chicago, Illinois, United States of America; 3 University of Pennsylvania School of Nursing, Philadelphia, Pennsylvania, United States of America; 4 The Corner Health Center, Ypsilanti, Michigan, United States of America; 5 University of California, San Francisco, California, United States of America; Brown University, UNITED STATES

## Abstract

**Background:**

Drug abuse and sexually transmitted infections (STIs), including the human immunodeficiency virus (HIV), remain significant public health concerns in the United States. Youth are at disproportionate risk of drug use and STIs/HIV, yet interventions aimed at improving STI and HIV testing and reducing STI/HIV risk behaviors through technology-based engagement in clinic settings are limited. The purpose of this study was to examine the feasibility and acceptability of Storytelling 4 Empowerment (S4E), a multilevel mobile-health drug abuse and STI/HIV preventive application (app) for clinic settings. We also explored uptake of STI/HIV testing among youth immediately post-intervention.

**Method:**

Employing community-based participatory research principles and a multi-method research design, we developed a clinician-facing app, and examined the feasibility and acceptability of S4E among clinicians (n = 6) and youth (n = 20) in an urban youth-centered community health clinic. S4E aimed to improve clinician–youth risk communication and youths’ drug use and STI/HIV knowledge, self-efficacy, and refusal skills. We also explored youths’ uptake of STI and HIV testing. Quantitative data were analyzed by computing mean scores and proportions, and qualitative analyses followed the tenets of content analysis.

**Results:**

Among eligible participants, 86.9% of youth and 85.7% of clinicians enrolled in the study, suggesting the feasibility of recruiting participants from the targeted clinic. Most clinicians identified as non-Hispanic white (83%) and female (66.7%). Among the youth, 70% identified as non-Hispanic white, followed by 30% African American, and 50% identified as female with a mean age of 19.6 (SD = 1.5, Range = 16–21). The quantitative findings suggest that the acceptability of S4E is high, as indicated by the Client Satisfaction Questionnaire (mean score = 25.2, SD: 4.8). Immediately post-intervention, all youth who reported past 90-day condomless sex or having never been tested for STIs or HIV in their lifetime, were tested for both STIs and HIV. Qualitative themes revealed four overarching themes, including S4E: (1) faciliated timely, targeted, and tailored prevention and risk reduction strategies; (2) shaped clinician and youth communication and interaction during the clinic visit; (3) may have improved uptake of STI/HIV testing and increased STI/HIV knowledge and self-efficacy; and (4) had high feasibiliy and acceptability among youth and clninicans.

**Conclusions:**

Findings suggest the feasibility and acceptability of S4E in an urban community-based health clinic setting. A next important step is to examine the efficacy of S4E in a randomized controlled trial design.

## Introduction

Drug abuse and sexually transmitted infections (STIs), including human immunodeficiency virus (HIV), remain significant public health concerns among youth in the United States [1–2]. For example, data from the Monitoring the Future study indicate that 33.2% of 12^th^ grade youth report past 30-day licit (i.e., alcohol) drug use, and 24.9% report illicit (i.e., other drug use, including the use of prescription pain medication without a doctor’s prescription) drug use [3]. The high rates of licit and illicit drug use among youth are troublesome because drug use behaviors enhance the vulnerability to engage in sexual risk behaviors, including condomless sex [4]. In fact, data from the Youth Risk Behaviors Surveillance Survey indicate that 49.9% of sexually active 12^th^ grade youth report condomless sex at last sexual intercourse [5], increasing their vulnerability to STI and HIV infection [6].

During 2014–2017, rates of STIs have sharply increased in the United States [7]. In 2016, more than 2 million cases of chlamydia, gonorrhea, and syphilis were reported in the United States. This is the highest number in history of the three nationally reported STIs [8]. Youth 15–24 years are disproportionately affected by STIs, accounting for 25% of the sexually active population and constituting 50% of all new STI diagnoses that occur in the United States each year [9]. The prevalence of STIs among youth is alarming, underscoring the importance of increasing STI screening and treatment routinely. Prior research has noted that risk factors linked to STIs often parallel key contributors to HIV acquisition and may also increase individuals’ biologic vulnerability to HIV acquisition if left untreated. [6]. Although the rates of HIV infection among youth aged 13–24 have decreased or remained stable between 2010 and 2014, they still account for a disproportionate number of new HIV infections each year. In fact, youth between the ages of 13–24 comprise approximately 16% of the total United States population, yet constitute nearly one quarter of all new HIV infections in the United States [10]. Importantly, it is estimated that nearly 60% of youth do not know they are infected with an STI, including HIV [11]. Therefore, improving uptake of STI and HIV testing has been identified as a key prevention strategy aimed at reducing transmission to uninfected partners while also improving linkages to STI/HIV prevention and care services [12]. The Centers for Disease Control and Prevention recommends annual STI and HIV testing in at-risk youth [13]. However, many youths are only tested based on their perceived risk, and few youths are routinely screened for asymptomatic STIs as recommended by the CDC [7]. Beyond inconsistent STI testing, national surveillance data indicate that 13.2% of youth report having ever been tested for HIV [5]. Taken together, these surveillance data highlight the critical need to develop and test developmentally and culturally congruent drug abuse and STI/HIV preventive interventions for youth.

Leveraging technology for prevention science is ideal given that the vast majority of the United States’ population has access to mobile devices, especially young populations [14]. Furthermore, youth-centered community health clinics are an ideal setting for delivering preventive interventions because many youth perceive these clinics as culturally congruent with their drug use and STI/HIV prevention needs [15]. Youth-centered initiatives align with federal recommendations focused on screening and treating youth’s drug use and sexual risk behaviors in clinic settings [13]. Despite the disproportionately high rates of drug use, sexual risk behaviors, and STI and HIV infection, as well as low rates of STI and HIV testing among youth, few technology-based interventions have been developed and shown to be feasible and acceptable for this population in a clinic setting [16].

To advance scientific knowledge on technology-based scalable solutions, we employed community-based participatory research (CBPR) principles [17] in conjunction with prevention principles [18] to develop Storytelling 4 Empowerment (S4E), a targeted and tailored mobile-health (mHealth) drug abuse and STI/HIV native application (app) [19]. S4E is theory-driven and guided by empowerment [20] and ecodevelopmental [21] theories. From an empowerment perspective, youth have the necessary strengths, capacity, and skillset to overcome drug use and sexual risk-related challenges, which may be enhanced by linking youth with important adult figures such as clinicians [22]. In line with ecodevelopmental theory [23], youth and their environment are shaped by one another in a reciprocally influential manner with a focus on adolescence and youth adulthood developmental periods [24]. These processes occur in systems, and the S4E approach is particularly concerned with the health clinic system. Guided by this framework, S4E aims to improve youths’ drug use and STI/HIV knowledge, self-efficacy, and refusal skills and youth-clinician communication. This is accomplished through a youth-facing native app, which consists of a brief risk behavior survey tool used to assess drug use and sexual risk behaviors and then provides targeted and tailored content based on the youth’s specific risk behaviors. The content, which is interactive, read aloud, and focuses on improving the potential mechanisms of change (i.e., youth–clinician communication, youths’ drug use and STI/HIV knowledge, self-efficacy, and refusal skills), provides culturally congruent storytelling scenarios that were developed with youth [19]. In previous research, youths’ usability and acceptability of the S4E user experience and user interface was high [25]. However, youth expressed their desire to engage in risk communication with their clinicians and for clinicians to initiate these challenging conversations [26].

Building on this formative research, we developed a clinician-facing native app as part of the S4E preventive intervention. The clinician-facing app provides clinicians with youth risk assessment responses, a risk communication interviewing tool kit (e.g., reflective questioning, motivational interviewing), and resources to link youth with prevention and care services. More specifically, clinicians are provided with youths’ responses to the drug use and sexual risk assessment, which are then used to facilitate tailored youth–clinician risk communication (e.g., drug use prior to sex, condomless sex) and linkage to targeted resources (e.g., HIV testing), as well as to reinforce prevention and risk reduction messaging that the youth received on their youth-facing app (e.g., drug use refusal skills). Taken together, the S4E preventive intervention aims to improve uptake of STI and HIV testing, and ameliorate drug use and sexual risk behaviors through a multilevel approach, focusing on intrapersonal (knowledge, self-efficacy, refusal skills) and interpersonal (youth–clinician risk communication) levels as a potential scalable solution to drug use and STI/HIV. Now that the S4E content has demonstrated high usability and acceptability among youth, a next important step is to determine the feasibility and acceptability of the updated version of S4E among youth and clinicians.

The purpose of the present study was to examine the feasibility and acceptability of S4E among *both* youth and clinicians in a youth-centered community health clinic. For the purpose of this study, we define feasibility as the enrollment rate (% of eligible youth and clinicians who were enrolled), intervention completion rates (% of clinicians and youth who completed all intervention activities or partial intervention activities), and clinicians’ acceptability of S4E (youths’ high acceptability of S4E has been reported elsewhere; [25]). In addition to determining the feasibility and acceptability, we also explored youths’ uptake of STI and HIV testing immediately post-intervention. We also collected secondary outcomes including drug use and sexual risk behavior. Additionally, through our qualitative interviews, we explored potential mechanisms of change of the S4E intervention, including youth–clinician risk communication and youths’ drug use and STI/HIV knowledge, self-efficacy, and refusal skills.

## Methods

### Ethics statement

Research Ethics Board approval (HUM00105293) was obtained from the University of Michigan, Ann Arbor, United States. A Certificate of Confidentiality was obtained from the National Institute of Mental Health.

### Intervention development

Our community–university formative research prompted us to develop a clinician-facing native app as part of the S4E approach [19]. We employed principles of community-based participatory research (CBPR) [17] in combination with design thinking [27] to inform the development of the clinician-facing app. CBPR and design thinking have complementary goals. CBPR principles aim to involve community partners in all aspects of the research process (e.g., identification of the problem, selection of intervention) [17], and design thinking aims to foster innovation through multidisciplinary teams to address health behaviors through a problem finding, problem selecting, solution finding, and solution selecting process [27].

In our study, the Youth Leadership Council (YLC), a diverse youth-led group focused on community health research and advocacy in the clinic, as well as clinicians from the participating youth-centered community clinic, have guided and continue to guide this program of technology-based preventive intervention research [19]. Our collaboration with the YLC and clinicians aimed to ensure that the intervention was culturally congruent for the targeted community and to improve intervention uptake. The study team attended weekly YLC meetings composed of 8–10 youth diverse in race/ethnicity, gender, sexuality and between the ages of 13–22, during a 6-month period. During these meetings, youth reviewed and decided on content and designs that were culturally congruent for the clinic youth population. As part of a larger study, clinicians identified a critical need to develop technology to highlight youth risk behaviors in a culturally congruent approach such as aligning with the clinic’s workflow (e.g., limited time with patients, no time to scroll through long risk assessments). To this end, we developed prototypes that included a risk assessment retrieval, motivational interviewing scripts to discuss risk behaviors, and referral resources to address the concerns raised by clinicians.

### Recruitment of participants

We employed a multi-method study design and recruited youth and clinicians between June 29 –September 14, 2016 from a youth-centered community clinic located in Southeast Michigan. To be eligible for this study, youth had to: (1) be between 13–21 years old; (2) live in Southeast Michigan; (3) have a scheduled appointment with a clinician enrolled in the study; and (4) report no prior history of psychiatric hospitalization. Recruitment of potential youth participants occurred during a clinic phone call reminder regarding their scheduled medical appointment. This recruitment strategy provided the research team member an opportunity to engage with youth prior to their visit, provide a brief overview of the study, answer questions, and address concerns. Recruitment of clinicians, including physicians, nurses, and social workers, occurred during a weekly clinic staff meeting. The study team provided an overview of the research and emphasized that participation in the study was voluntary. All clinicians at the targeted youth-centered community health clinic were eligible to participate in this study. To prevent coercion, clinicians were not required to go through administration to enroll in the study nor inform them of their participation in the study. Clinicians who expressed interest in participating in the study contacted the study team to enroll.

We attempted to contact a total of 44 youth who were eligible to participate in the study and had an appointment scheduled with an enrolled clinician. Of these, 14 youth did not return messages from the study team, six reported schedule conflicts, and four had disconnected phone numbers. The remaining 20 participants were enrolled in the study. Seven clinicians were approached during the clinic staff meeting, and six clinicians contacted the study team to participate and were subsequently enrolled in the study. Among youth (n = 20), 70% (n = 14) identified as non-Hispanic white, followed by 30% (n = 6) African American. Fifty percent (n = 10) of the youth identified as female, followed by 35% (n = 7) transgender and 15% (n = 3) male, with a mean age of 19.15 (SD: 1.56; Range: 16–21). The relatively high proportion of transgender youth may be partially explained by the youth-centered clinic’s inclusive services such as gender-affirming health care. Among clinicians (n = 6), 83% (n = 5) identified as non-Hispanic white, 66.7% (n = 4) female, and the mean age was 41.8 (SD: 13.2, Range: 28–65). Fifty percent of the clinicians reported a specialty in primary care medicine, and the average years of clinical experience was 14.2 (SD: 14.6, Range: 1–40).

### Procedures

According to Title X in conjunction with the Public Health Code, MCL 333.6121, a minor 17 years of age or younger can consent to sexual and reproductive health and substance use services in the state of Michigan. Therefore, to protect the confidentiality of youth who receive services at the clinic without parental knowledge, we obtained a waiver of parental consent. All youth and clinicians provided consent through a comprehensive written waiver of documentation without signature. Comprehensive refers to all of the elements of informed consent required by Health and Human Services regulations and policy are presented to participants. Waiver of documentation without signature refers to the use of a comprehensive consent process without obtaining a signature from the participant or legally authorized representative. As part of the consent process, youth were informed that their clinician would have access to their risk assessment responses in the S4E app. Youth and clinicians provided consent and were assessed at baseline and immediately post-intervention. Eligible youth arrived one hour prior to their scheduled clinic appointment to have the study explained to them, provide consent, complete baseline assessment, and participate in the S4E intervention via tablets in a reserved room located in the clinic. Data were collected via RedCap, a HIPAA-compliant, web-based application for research data capture [28]. Upon completing the youth-facing S4E intervention, youth met with their clinician for the clinician–youth prevention and risk reduction encounter. Additionally, youth participated in an individual interview at the completion of the post-intervention assessment, and clinicians participated in an individual interview once all 20 youth completed the study. Youth received $20 for their participation in the study, and the youth-centered community clinic received $2500 for clinicians’ participation in the study to benefit the whole clinic.

### S4E intervention

**Fig 1:**
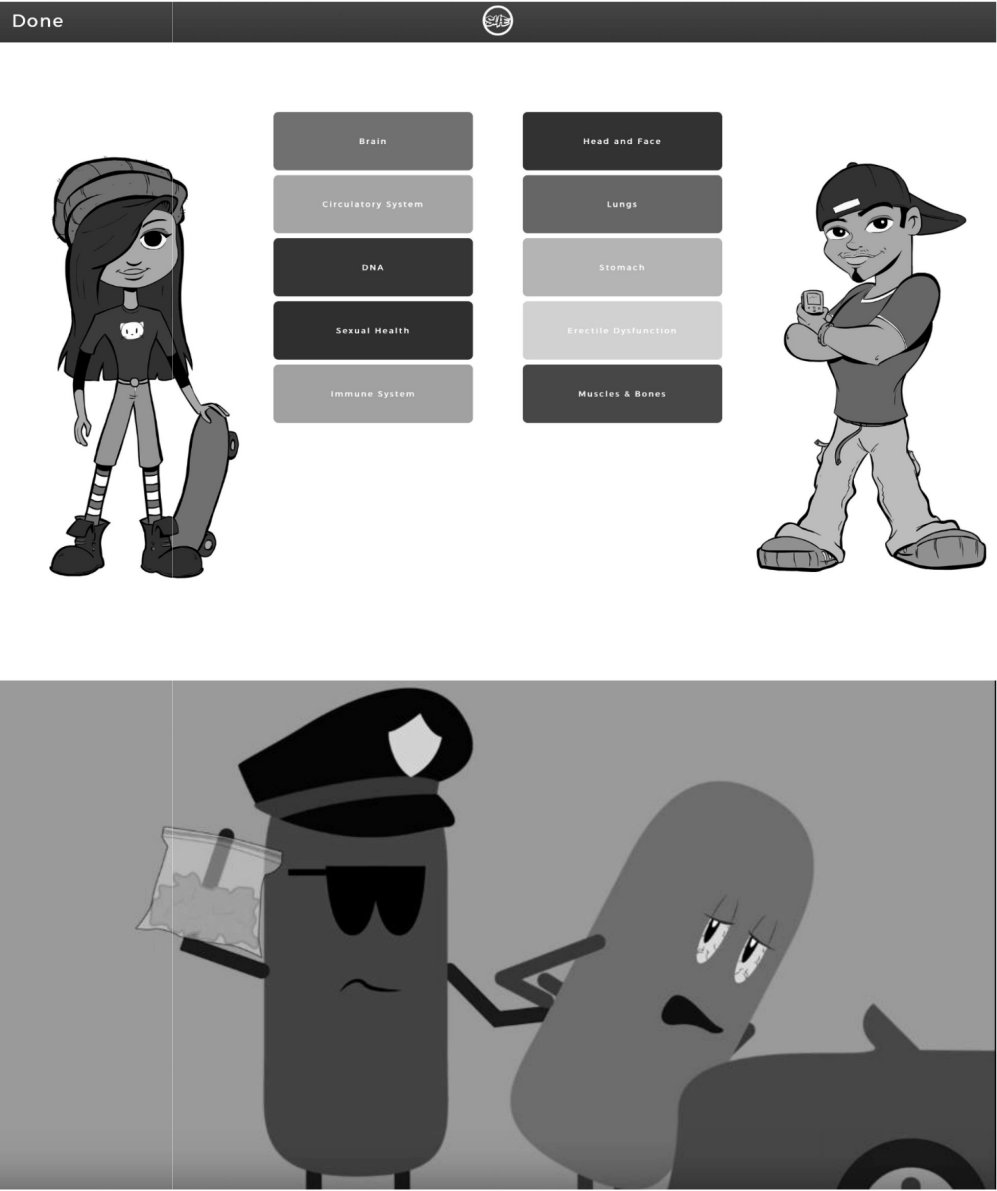
S4E youth prevention intervention content.

S4E aims to improve drug use and STI/HIV knowledge, self-efficacy, and drug use and sexual risk refusal skills, as well as clinician–youth risk communication through targeted and tailored interactive content. This is accomplished through three core components of the youth-facing app. First, youth complete a brief 20-question risk behavior assessment that assesses drug use—including the Car, Relax, Alone, Forget, Friends, Trouble (CRAFFT) [29]—as well as sexual risk behaviors and STI/HIV testing practices. Second, the youth’s responses to the brief S4E risk assessment are then used to deliver targeted and tailored prevention and risk reduction content. Specifically, the youth is provided with recommended content based on their specific risk behaviors, which consists of interactive activities, storytelling scenarios, psychoeducational material, and messaging focused on drug use and sexual risk prevention. Beyond the recommended content, the youth is granted access to additional prevention and risk reduction content. Finally, the youth is provided tailored resources based on their brief risk assessment responses to link them to prevention and care (Fig 1).

**Fig 2:**
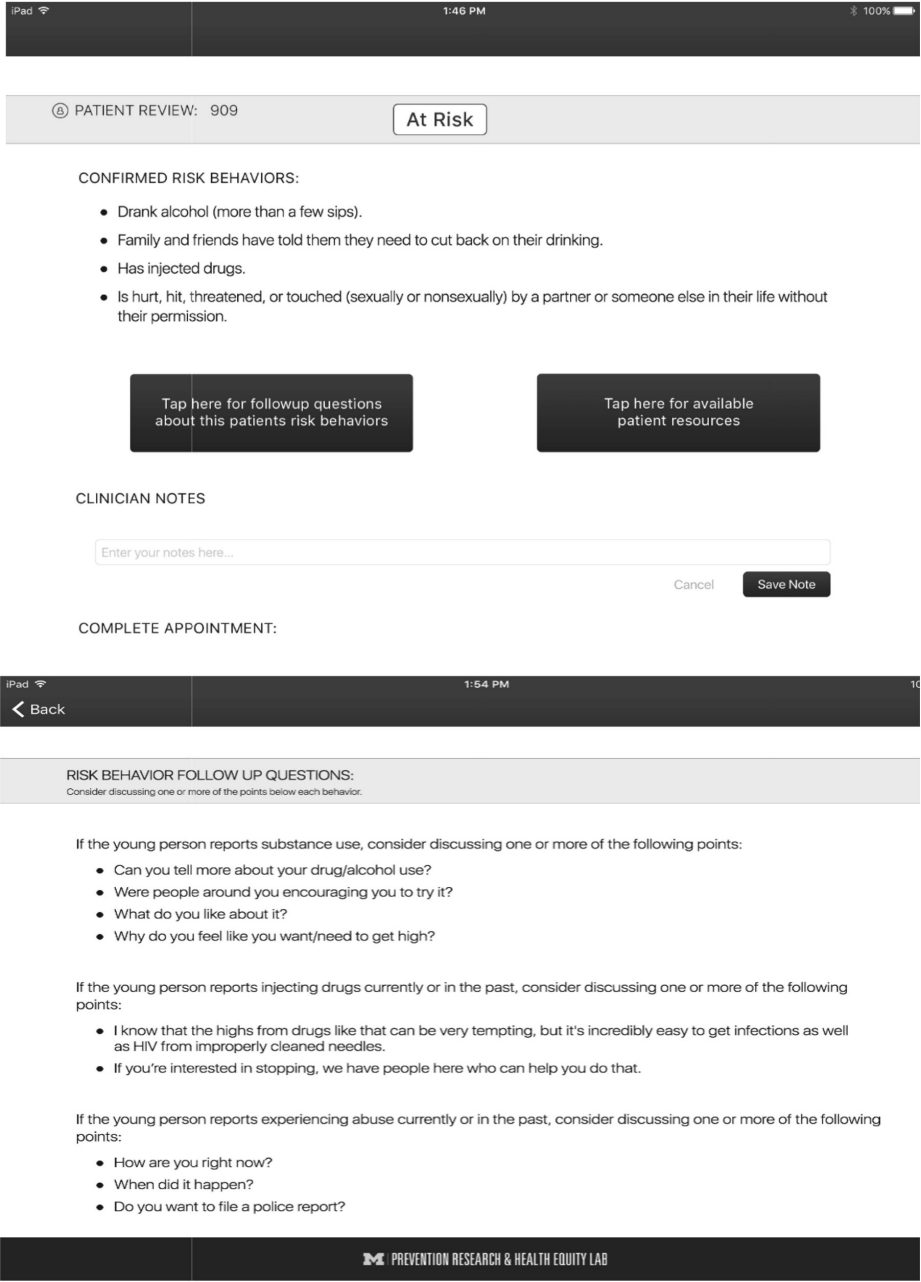
S4E clinician prevention intervention content.

The clinician-facing app provides clinicians with key resources to enhance clinician–youth communication about drug use and STI/HIV during the youth–clinician prevention and risk reduction encounter. This is achieved through three core components of the S4E clinician-facing app. First, youth risk assessment responses are provided to the clinicians via the app. Immediately before the clinician meets with the youth, they review the youth’s risk score and learn about their risk behaviors. This provides an opportunity for the clinician to deliver and reinforce targeted and tailored prevention and risk reduction strategies based on the youth’s specific risk behaviors. Second, the clinician can access a tailored risk communication toolkit (e.g., reflective questioning). This toolkit can help them facilitate conversations about risk behaviors and treatments. For example, if the youth responds “yes” to condomless sex and “no” to having received an HIV test, the clinician is provided with scripts to engage the youth in risk communication that highlights the benefits of STI/HIV testing and using condoms correctly and consistently. Finally, the clinician receives targeted resources, the same resources recommended to youth, as an opportunity to reinforce health promotion opportunities and link the youth to prevention and care (Fig 2). Taken together, S4E is a multilevel mHealth strategy that focuses on intrapersonal (i.e., drug use and STI/HIV knowledge, self-efficacy, drug use and sexual risk refusal skills) and interpersonal (i.e., clinician–youth risk communication) levels aimed at improving uptake of STI/HIV testing, and ameliorating drug use and sexual risk behaviors among youth.

### Quantitative measures

#### Demographics (completed at baseline)

Youth and clinicians completed a demographic survey that asked their gender identity, sexual orientation, age, ethnicity/race, and education. Clinicians also reported their medical specialty and years of clinical experience.

#### Drug use and sexual risk behaviors (completed at baseline)

We assessed youth’s licit (i.e., alcohol) and illicit (i.e., other drug use, including prescription pain medication without a doctor’s prescription) drug use behaviors using items adapted from the Monitoring the Future study [3]. Youth were asked whether they had used licit or illicit drugs in their lifetime and in the past 90 days prior to the assessment.

We assessed youth’s sexual risk behaviors using items from the Sexual Behavior instrument [30]. This measure is gated such that youth who reported not having sex in their lifetime were not asked about past 90-day sexual risk behaviors, nor age of sex initiation (“When you first had vaginal, anal, or oral sex, how old were you?”) nor lifetime and past 90-day condom use (“In the past 90 days, about how often have you had vaginal or anal sex without using a condom?”). This measure also assesses STI and HIV status during their lifetime and in the past 90 days (“Has a doctor or other health care professional ever told you that you had a sexually transmitted infection?”). Youth who reported “yes” to past 90-day sex were also asked to report any drug use prior to sex (“During the past 90 days, have you been under the influence of alcohol before having sex?”). For the present study, all drug use and sexual risk behaviors were coded as binary variables.

#### STI and HIV testing (baseline and immediately post-intervention)

We assessed STI and HIV testing among youth using four items. Youth were asked, “Did you receive an HIV test?,” and “Did you receive a STI test?” Additionally, clinicians were asked, “Did your patient receive an HIV test during their visit?,” and “Did your patient receive a STI test during their visit?”

#### Clinician–youth communication (immediately post-intervention)

We assessed clinicians’ (α = 0.96, 19 items) and youths’ (α = 0.99, 19 items) experiences related to clinician–youth communication using items adapted from the Matched Pair Instrument (MPI) [31]. The MPI assesses process and content of communication, including verbal and action-related behaviors performed by clinicians. Using a five-point Likert scale, responses range from “1 = strongly disagree” to “5 = strongly agree.” A sample statement for clinicians is, “Encouraged the patient to express his or her thoughts concerning drug use and/or sexual risk behaviors.” A sample statement for youth is, “Encouraged me to express my thoughts concerning drug use and/or sexual risk behaviors.” Ratings of 4 and 5 indicate satisfaction with the clinician’s communication skills [31].

#### Acceptability (post-intervention)

We assessed clinicians’ acceptability of S4E using an adapted Client Satisfaction Questionnaire-8 (CSQ; α = 0.91) [32]. The CSQ-8 consists of eight items and assesses clinicians’ overall satisfaction with the S4E intervention. An example question is, “If a colleague were in need of a similar tool, would you recommend the app to him or her?” Response options range from “1 = No, definitely not” to “4 = Yes, definitely.” A cumulative CSQ score was created by adding each participant’s score on each item, resulting in a maximum score of 32. A higher score on the CSQ indicates greater satisfaction [32].

### Quantitative data analysis

The analysis proceeded in three steps. First, we conducted a descriptive statistics analysis for demographics and outcome variables to characterize the sample. Second, to test intervention acceptability by clinicians, we computed the mean acceptability scores for the CSQ-8. Finally, we explored uptake of STI and HIV testing among youth by observing changes in the proportions of STI and HIV testing endorsement at baseline and immediately post-intervention. All data were analyzed using SPSS Version 25 [33]. Consistent with the formative nature of our feasibility and acceptability study, the primary goal of our study was to estimate the critical parameters required to note whether our intervention had sufficient acceptability and feasibility in preparation for a preliminary efficacy trial [34–37]. As a result, we were not powered to estimate small effect sizes (i.e., baseline vs immediately post-intervention) or carry out sophisticated statistical analyses (i.e. mediation).

### Qualitative measure

The interview guide for both clinicians and youth interviews consisted of a series of open-ended questions. Questions in the youth interview guide included “What was your experience participating in the intervention using the S4E app?” and “Please describe your experience communicating with your clinician during this particular office visit.” Questions in the clinician guide included “What are your overall thoughts regarding the risk assessment after having participated in the program?”, “What did you like best about the app?”, and “Please describe your experience communicating with your patient while using the app.” Individual interviews were audio-recorded and conducted in private rooms in the clinic setting by study team members. The interview guide was modified after each individual interview to include additional probing questions identified during previous interviews. Youth interviews occurred after the clinic visit, and clinician interviews occurred at the end of the intervention and ranged in total time from 20–40 minutes for youth and 15–30 minutes for clinicians.

### Qualitative data analysis

Audio recordings for each interview were transcribed verbatim and reviewed for accuracy by multiple study team members. Data were uploaded to Nvivo10 [38] for storage, organization, and data analysis. Data were analyzed through a content analysis consisting of systematically condensing the data into related subcategories, categories, and overarching themes. First, three members of the study team, including the lead author and two research assistants, read each transcript to familiarize themselves with the data. Second, the team conducted open coding with respect to the overarching research question: Is S4E feasible and acceptable among clinicians and youth? Third, the codes were collated into emerging subcategories (e.g., clinicians expressed positive experiences related to prevention and risk reduction encounters with youth). Fourth, the team merged related subcategories to major categories and themes (e.g., clinicians reported high feasibility and acceptability of the intervention). The study team determined trustworthiness of data through credibility, transferability, and dependability [39]. For example, we determined credibility through member checks with the YLC and clinicians during meetings. Additionally, prolonged time was spent in the clinic with staff and clinicians to ensure transferability. Lastly, we determined dependability through careful tracking of the research activities and processes, including the research design (e.g., multi-method), data collection (e.g., asking youth to arrive one hour prior to their appointment), and identifying emerging themes (e.g., meeting with independent coders to discuss emerging themes) [39].

## Results

### Quantitative

As shown in Table 1, among youth who reported lifetime licit and illicit drug use, the majority of youth reported past 90-day licit (78.6%, n = 11) and illicit (50.0%, n = 4) drug use. Among the 11 participants who reported past 90-day vaginal, anal, or oral sex, 63.6% (n = 7) endorsed condomless vaginal or anal sex, and 81.8% (n = 9) reported condomless oral sex in the past 90 days. Approximately 50% (n = 10) of youth received a score of 2 or more on the CRAFFT [29]. Furthermore, 26.3% (n = 5) of youth reported an STI diagnosis in their lifetime.

**Table 1:** Youth risk behaviors.

Variable	Frequency	% (M/SD)
Lifetime alcohol use (n = 20)	16	80
Past 90-day alcohol use (n = 14)	11	78.6
Past 90-day binge drinking (n = 11)	3	27.3
Lifetime illicit drug use (n = 19)	11	57.9
Past 90-day illicit drug use (n = 8)	4	50
Age of sex initiation (n = 17)	15	(15.3 / 2.4)
Lifetime vaginal, anal, or oral sex (n = 19)	17	89.5
Past 90-day vaginal, anal, or oral sex (n = 17)	11	64.7
Lifetime condomless anal or vaginal sex (n = 17)	14	82.3
Past 90-day condomless anal or vaginal sex (n = 11)	7	63.6
Lifetime condomless oral sex (n = 17)	17	100
Past 90-day condomless oral sex (n = 11)	9	81.8
Lifetime alcohol use before sex (n = 17)	10	58.8
Past 90-day alcohol use before sex (n = 11)	5	45.4
Lifetime drug use before sex (n = 17)	2	11.8
Past 90-days drug use before sex (n = 11)	2	18.2
Lifetime STI diagnosis (n = 19)	5	26.3
Past 90-day STI diagnosis (n = 5)	1	20
CRAFFT At-Risk (score 2+)	10	(3.6 / 0.84)

At baseline, 50% (n = 10) of youth reported having received an STI or HIV test in the past six months. Immediately post-intervention, all youth who reported past 90-day condomless vaginal or anal sex or having never been tested for STIs or HIV in their lifetime, were tested for both STIs and HIV. Specifically, 35% (n = 7) of youth received both an STI and HIV test. Among the youth tested immediately post-intervention, 85.7% (n = 6) reported condomless oral, vaginal, or anal sex in the past 90 days, and 14.3% (n = 1) had never been tested for an STI or HIV in their lifetime.

We also explored clinician–youth communication, a potential pathway through which change occurs. Clinicians’ mean Matched Pair Instrument [31] score was 4.12 (SD: 0.72), and for youth was 4.18 (SD: 0.90). Clinicians’ mean Client Satisfaction Questionnaire [32] score was 25.2 (SD: 4.8, Range: 17–29), suggesting high acceptability of the S4E intervention.

### Qualitative results

The primary goal of the present study was to examine the feasibility and acceptability of the S4E intervention. The main themes identified are: (1) S4E faciliated timely, targeted, and tailored prevention and risk reduction strategies; (2) S4E shaped clinician and youth communication and interaction during the clinic visit; (3) Uptake of STI/HIV testing and increased STI/HIV knowledge and self-efficacy; and (4) clinicians and youth reported high feasibility and acceptability of the S4E intervention (Table 2).

**Table 2:** Qualitative results.

Themes
**S4E facilitated timely, targeted, and tailored prevention and risk reduction strategies.**
Clinicians
• “I think it’s really great especially for adolescents to get that immediate feedback in the forms of the videos and the intervention. So, it’s not like they filled out the questionnaire and two weeks later somebody said, ‘Hey, let’s give you some information about smoking cessation.’ They got it immediately.” (Clinician 5)
• “I like that it pops up with risk factors and saying what we need to be talking about or looking out for.” (Clinician 2)
• “When I walked into the room, she was watching the [intervention] videos, so I knew this was something that we could talk about.” (Clinician 4)
• “I really liked that it sorted out things that need to be addressed. In our system, you have to scroll through everything to find a patient’s risk. It helped me focus on the relevant.” (Clinician 4)
• “I said, ‘You’ve had unprotected sex. You haven’t always used condoms?’ And they said, ‘Yeah, this is why; this is what I hope to do in the future.’”(Clinician 1)
Youth
• “If it were a test, I would feel well studied…it’d be like reviewing a sheet that you’ve done all of your test prepared on before you actually take the test. So, it just reminds you of what you can talk about, if that makes sense at all.” (18, Trans male, White)
• “That actually made it easier cuz I put my problems in and then he looked at it and knew what my visit was for, so it made it easier.” (20, female, Black)
• “I liked it [S4E] because it wasn’t like I had to explain it [risk behaviors] over and over and over again. They [clinician] already know. So, I don’t have to explain it.” (18, female, White)
**S4E shaped clinician and youth communication and interaction during the clinic visit.**
Clinicians
• “Well, one thing that helped is that I knew that the patient wanted to talk about it and it's probably not a cold call. Like, you know that they're already somewhat open to talking about it so in that way that makes it a little bit easier to bring it up.” (Clinician 4)
• “It facilitated conversations between me and my patients about their risk behaviors, which I really celebrated it for that. Even though I feel like my patients are pretty open with me, it definitely allowed us to, even if we've had that conversation before, to just take a step back and say, ‘Oh, by the way, you know this app tells me that one of your risk behaviors is this.’” (Clinician 3)
• “So, I was able to just quickly and honestly address risk behaviors in a way that was non-threatening.” (Clinician 3)
• “It's easier to put yes on an iPad than to say it out loud.” (Clinician 2)
Youth
• “We had nothing else to talk about, and she just like pulled up the app, and she was like going through it, and she was like, ‘Are you okay if we talk about this?’ and I was like, ‘Yeah!’” (19, male, White)
• “Mainly because I have a trust issue. It’s hard for me to trust people so I can’t just up and tell someone about my past and with her, I know that I can tell her anything. We brought it up, and she knows stuff that my boyfriend doesn’t even know.” (19, male, White)
• “We have the plan to like, ‘What do you want to focus on, this?’ So, like, I think this app allowed that like focus to be brought out.” (20, female, White)
• “It helped because it helped him understand the type of person I am and it got him to where he would be able to ask me questions to break down who I actually was.” (21, male, Black)
• “We have the plan to like, ‘What do you want to focus on?’ So, this app allowed that focus to be brought out.” (20, Trans female, White)
• “I think it [S4E] brought us a little closer cuz I never discussed drugs or anything with her.” (16, female, White)
**Uptake of STI/HIV testing and increased STI/HIV knowledge and self-efficacy.**
Clinicians
• “I had some that were curious about it like, “Ok, so, if I wanted to do that, would that involve, or, you know, actually making an appointment?” Just kind of like questions about like if they considered it in the future, how would they go about doing it.” (Clinician 2)
Youth
• “I would’ve gotten another one [HIV Test], but I was tested in [date], and I haven’t had sex since.” (17, female, Black)
• “It’s not something I’m particularly worried about. But I haven’t had a test before, and maybe I should do that sometime. She was bringing it up because I said I hadn’t gotten tested, so like, ‘Would you like to?’ And I said, ‘Sure.’ I think it did help facilitate that.” (18, Trans male, White)
• “It was helpful, especially about the alcohol and I would say marijuana. Like being in college, and those are the two main things I hear about … So, it was good to know facts about that.” (20, female, Black)
• “It gave a lot of information and insight about STD’s and STI’s, drug use, alcohol use and things of that nature that was very helpful.” (20, Trans female, Black)
**Clinicians and youth reported high feasibility and acceptability of the S4E intervention.**
Clinicians
• “Well I think the content, the way it was organized, and worded was very substantive and good.” (Clinician 6)
• “I thought it was very self-explanatory, very user friendly.” (Clinician 5)
• “I found it easier than a traditional model of electronic risk assessment. Because I knew that they’d already had feedback from the intervention.” (Clinician 5)
• “It wasn’t like I had to learn a whole new communication skill set. And I didn’t have to incorporate anything new into my visit.” (Clinician 4)
• “It fills time in the clinic visit that would’ve been otherwise wasted, with something that, in my mind, is very productive.” (Clinician 2)
• “The app gave us a third party to put the burden of the emotional baggage on. You know, it wasn't us talking about, ‘So tell me about your sexual behaviors.’ It was like, ‘No, the app tells me that you've had some risky sex. You wanna talk to me about that?’ So, I felt like it was a really great tool in terms of that.” (Clinician 3)
• “It saved me time in terms of what I needed to cover. When time is limited, I can hone right into the things that the patient is worried about.” (Clinician 3)
Youth
• “I was really satisfied. I just like it. Coming to things and having a lot of things to do keeps me feeling I’m important.” (18, Trans male, White)
• “It was pretty satisfying, mostly satisfied.” (21, female, White)
“Very satisfied. Like helping talk about things during the visit that I wasn’t previously planning on talking about, definitely pushed the conversation along.” (18, male, White)
• “I know that while watching it [intervention videos] just felt like information was just being thrown at you in a way that made sense. Like the ‘fast facts’ was really interesting cuz it kept it short and sweet and right to the point. And it elaborated on a lot of stuff. And then some of the videos, it was just fun and kind of crude in a way too that made you keep interest in it. So, I feel a lot of the stuff, even if I got an answer wrong [interactive activity], I still retained.” (20, female, White)

#### S4E facilitated timely, targeted, and tailored prevention and risk reduction strategies

*S4E provided youth time to prepare for their clinic visit and discuss risk behaviors (n = 16)*. Youth completed a risk behavior assessment that would be shared with their clinician prior to receiving access to the S4E intervention content. This risk assessment was an opportunity for youth to reflect, prepared youth to engage in conversations and disclose risk behaviors, as well as for clinicians to have access to real-time youth health information. For example, one youth shared, “I knew what the questions he would ask me would be, because I saw them and answered them first. It wasn’t like face-to-face beforehand. I got to prepare for the conversation” (18, Trans male, White). Another youth reflected on how their responses to the risk assessment brought attention to risk behaviors that they were not intending to discuss during their clinical encounter:

It guided the conversation to things that I might not have brought up myself. I wasn’t really sure if I was going to talk about drugs or alcohol at all today and for the most part, it was unrelated to that. I was sort of like, ‘Well this isn’t really like what’s most important to me right now,’ so I wasn’t going to talk about it. But I think, you know, he definitely thought it was helpful to talk about. (18, Trans male, White)

Additionally, clinicians found that S4E offered a timely opportunity for youth to anticipate and discuss prevention and risk reduction strategies with them. One clinician described:

When using a more traditional questionnaire model, the patient answers the questions. And when I get in the room, the patient doesn’t have any baseline knowledge about what might happen next based on their risk. But with the app, my experience was more the patient could anticipate what I was going to say. (Clinician 5)

*S4E facilitated targeted and tailored prevention and risk reduction strategies (n = 5)*.Clinicians found that the S4E intervention provided an optimal tool that helped identify risk behaviors in a timely manner. One clinician expressed, “The way it was presented to the clinicians was very easy to focus on the important things. I didn't have to go through many questions and pick out the things that were important. The app did that for us. I really appreciated that” (Clinician 3).

By providing clinicians an efficient way to identify youth’s specific risk behaviors, clinicians were able to deliver targeted and tailored prevention and risk reduction strategies. One clinician described using the risk behavior survey to engage in meaningful conversations focused on sexual risk behaviors:

Based on their risk factors I would ask them what they do to protect themselves. I had a client who says him, and his partner were tested. And he trusts his partner, and he’s just not gonna get tested. So, we talk about that, we talk about what trust means, and we talk about how you have to be in control of your own body regardless of what somebody else tells you. But, if you are going to participate in that, then how can you continue to protect yourself, ‘Do you think you should still get tested every six months?’ (Clinician 1)

#### S4E shaped clinician and youth communication and interaction during the clinic visit

*Youth described how the intervention helped facilitate the clinician–youth encounter (n = 17)*. Youth expressed that the S4E intervention shaped their conversations and ensured concerns were addressed with their clinician. For example, one youth shared:

It was very structured, and I really like that just to try to make sure everything is talked about. Usually when I go to appointments it feels like I always come out and not talked about one thing that I wanted to talk about. So, this allowed me to at least have like equal time. (18, female, White)

*Clinicians expressed that the intervention shaped risk communication in meaningful ways (n = 6)*.Clinicians identified the ways in which the S4E intervention helped shape how youth discussed their risk behaviors during instances when the youth may not be as forthcoming about their risk behaviors. One clinician described:

I can just say [to the patient], “This is what it's [app] telling me.” And I feel like patients are a little more honest. They're primed to be a little more honest when they already said that [reponse to risk behavior assessment]. One of the patients I was talking to, I was like, “So, do you use condoms?” And they're like, “Yeah, I use condoms.” But after the app, I was like, “So, the app says that you haven't used condoms every time.” And they’re like, ‘Yeah, well there was this one partner where we decided not to.’ So, I feel like it definitely leads to a different type of answer. (Clinician 3)

In addition to youth risk behavior responses, the clinician can access a tailored risk communication toolkit (e.g., reflective questioning). This toolkit helps clinicians facilitate conversations about risk behaviors and treatments. Clinicans reported that the communiation tool kit had great utility as a resource when engaging youth in conversations related to risk behaviors. One clinician shared:

There was definitely a time where a risk factor popped up in the app and I was like, ‘Uh, how do I start a conversation about that?’ So, I went in [communication tool kit], here's some ways to start a conversation. I got some ideas to start that conversation and then I was able to do that. (Clinician 3)

*Youth reported improvements in the relationship with their clincian (n = 4)*. Youth expressed that participating in S4E and being encouraged to disclose risk behaviors through the intervention resulted in improved relationship with their clinician. Consequently, youth expressed that they would be more inclined to disclose risk behaviors during future visits:

I think it’s [relationship] become stronger actually. She knows more about me [because of my respones to the app risk risk assessment] that I didn’t even think to bring up. In future visits, I feel a lot more could happen, that I could bring up to her. I feel more, I know that she knows that part of my life. I can bring up more stuff that’s happened if I remember it and other things that have occurred. (18, male, White)

Some clinicians found that the intervention only helped achieve a deeper connection with patients with whom they were not familiar: “It was the second time I've seen them, so, basically a stranger, but I still had that hour that we had talked the time before. And it definitely allowed us to get deeper into their personal life quicker than we would have otherwise” (Clinician 3).

#### Uptake of HIV testing and increased STI/HIV knowledge and self-efficacy

*Youths’ and clinicians’ perceived increase of STI/HIV prevention knowledge and self-efficacy (n = 12)*. Clinicians indicated that the intervention may have improved youths’ STI/HIV knowledge and self-efficacy, which shaped their communication and the content that was discussed during the visit. One clinician described:

A patient, I’m assuming from the intervention that they did on the app, was familiar with what I was saying. I felt like there was less explaining I needed to do. In one instance, the patient even brought up themselves syphilis testing before I got to it. I thought that was pretty cool. They must have learned that from the app…I can’t imagine they came in thinking they wanted syphilis testing. (Clinician 5)

Similarly, youth reported that the S4E intervention content provided new STI/HIV prevention knowledge and self-efficacy for STI/HIV testing that they would have otherwise not received during their clinic visit. One youth shared how this knowledge may have been applied in previous relationships by encouraging the need for STI/HIV testing:

There were actually a lot of STI and STD-related things that I didn’t necessarily know were related to STI and STDs. They talked about in the app in the short videos. There’s actually been circumstances where I have known people in the past that I could have suggested that they got tested or something. (18, Trans male, White)

*S4E faciliatied STI and HIV testing and substance use communication (n = 17)*. Youth expressed that their participation in the intervention facilitated their conversations with their clinician regarding sexual risk and drug use behaviors. One participant reflected on their experience:

I would say it would be different. I wouldn’t have spoke up about alcohol or unprotected sex as often cuz its weird to talk to an adult about it. It helped a little bit taking to make sure I don’t have those things and to get tested and stuff. (20, female, Black)

Youth also mentioned that disclosing their risk behaviors was easier via the intervention, as compared to traditional face-to-face approaches. One youth described:

Things like drug and alcohol abuse are something that’s really uncomfortable to talk about. It’s a lot easier, especially I think for young people, to talk about things digitally. I think that’s why it’s a lot easier to talk over text than it is over the phone. It’s a lot easier to bring up problems that might be had on an iPad screen and then talk about it later in-person, rather than having to say it yourself [first] in-person. (18, male, White)

*The intervention helped improve uptake of STI/HIV Testing (n = 9)*. Participants shared the ways in which the intervention improved their willingness to uptake STI/HIV testing. For example, one youth shared, “I can definitely see it [the intervention] being useful. Personally, it made me decide to get an HIV test, so might as well do that” (18, Trans male, White).

Some participants revealed that they used other forms of birth control (other than condoms) and were in a relationship, and therefore did not perceive risk for STI and HIV infection. However, clinicians communicated to youth that they can never be certain, which may have improved uptake of STI and HIV testing. One youth reflected:

I’m on birth control and we've both already been tested for STDs and everything. So, she [clinician] said, “Do you know for sure that he's only sleeping with you?” And I was like, “You know, you're never sure.” (21, female, White)

Additionally, the intervention helped clinicians to highlight to youth STI/HIV risk behaviors that the youth had reported and to recommend STI/HIV testing. For example, one clinician described:

I was able to say, “Sounds like you've had some risky behaviors and I see that you haven't had an HIV test. Would you be interested in getting one?” And this patient scheduled themselves for an HIV test. (Clinician 3)

#### Clinicians and youth reported high feasibility and acceptability of the S4E intervention

*The feasibility and acceptability of S4E among clinicians was high (n = 6)*. Clinicians expressed that delivering S4E in a clinic setting is feasible, especially as it relates to clinic flow processes. One clinician shared, “I think the process moved pretty well. It didn't seem to be any longer than it takes our patients to fill out the risk assessment or the social history that we have now” (Clinician 4). Furthermore, clinicians expressed the importance of considering time when implementing interventions, as interventions may disrupt the clinic flow. One clinician shared that the S4E intervention’s process was feasible to the clinic setting:

There is a concern about the time it will take and the clinic flow. We do not want to add to the time it takes to get a patient roomed, add anything to the MAs’ or receptionists' plate. I thought that whole process was quite good actually. I have no qualms with it once I got trained and figured it out. (Clinician 3)

More specifically, clinicians found the parallel design of the intervention was a feasible way to deliver prevention and risk reduction strategies without interrupting the clinic flow. For example, one clinician described:

It was perfect. The patient did the risk assessment and was doing their responses [to the risk assessment]. While they were doing the intervention part, I was reviewing their responses. And the timing worked out such that they were finishing up the intervention as I came into the room, so it couldn’t have been better. (Clinician 5)

Furthermore, clinicians shared that the S4E intervention provided prevention strategies that may have not otherwise been shared due to time constraints. One clinician shared:

It took some of the burden off my visit because we didn’t have a lot of time and there were a lot of things to talk about. The patient had some baseline intervention via the app before I even got in the room. As much as I think I’d do a really good job with communication, you forget things. You leave things out. And the app doesn’t forget things. (Clinician 5)

Lastly, clinicians discussed the acceptability of the user experience and interface. One clinician mentioned, “When the technology cooperated, it was fine. It was pretty simple logging in and pulling up their info and just discussing that” (Clinician 2). Similarly, another clinician shared, “I thought it [S4E] was very self-explanatory, very user friendly” (Clinician 5). Additionally, clinicians shared their experience using a mobile device to deliver an intervention in a clinic setting. One clinician described, “S4E is more portable than sitting at a desktop. And better than a written paper. I think it’s a good idea” (Clinician 1).

Clinicians also shared ideas for potential improvements to S4E. For example, clinicians described potential usability improvements that could be made to the intervention to make the process of integrating technology more acceptable to the clinic and its workflow. For example, clinicians recommend incorporating the intervention into the clinic’s electronic medical system (EMR) to reduce the number of platforms they review for the patient. One clinician described:

My dream would be that this would feed right into the EMR. That the patient would still, in my dream world, do the app on their tablet or whatever they were using. But the risk behaviors would load automatically into the EMR so that when I open to their chart, I would see their risks based on their completion of the app. (Clinician 5)

Additionally, clinicians also recommended being able to access the intervention content with which youth engage in order to inform the clinicians’ conversations with their patients. For example, one clinician described:

One of the things I found challenging was not having the full risk assessment. So, ideally we would have the full questionnaire with everything they answered and then also, here are specific areas in which you might want to focus. It would show the areas of concern and what questions they answered in what way to cause the app to generate the risk behavior. (Clinician 3)

*Youths perceive the S4E intervention is highly feasible and acceptable (n = 17)*Youth affirmed that participating in the S4E intervention prior to their appointment was feasible. One youth expressed, “Participating in [S4E] was cool, it didn't take too much out of my day. And I had the opportunity to say no or be like this is taking too long so that was cool too” (21, female, White).

Additionally, S4E’s multilevel approach whereby youth engage in the intervention and then immediately participate in a clinic-initiated encounter was found feasible to reinforce prevention and risk reduction messaging. One youth expressed,

It made me more aware and alert of my answers and talking about them made me think more objectively than subjectively. It really clears things up. It puts things into a better perspective, and you're able to determine what’s right and what’s wrong more distinguishly instead of like as little small parts. It really brings it all together, so you can think about it as a whole. (17, female, White)

Additionally, youth found the S4E intervention acceptable because the content was presented in an innovative manner that was different from how they are accustomed to viewing prevention programs. For example, one youth mentioned:

I just feel like it [S4E] was really informational without getting boring. I know it’s a really big thing that stuff that can be informational and helpful can get really boring, but it was nice. It kept a good pace, and I liked it a lot. (18, female, White)

Youth also shared that they would recommend the S4E intervention to their friends, another indicator of acceptability. One youth mentioned, “I would recommend it [S4E] because it was really informative” (18, Trans male, white). Another youth shared, “Like my best friends, to know my experience and what I learned from it [S4E]. I’m pretty sure they’ll want to [participate]” (20, female, Black).

Participants also shared suggestions for how to improve the process of participating in the S4E preventive intervention. Some youth found it challenging to complete all intervention modules during their appointment and suggested providing access to S4E outside of the clinic setting. For example, one youth expressed: “The only thing I would say is maybe if somebody wanted to complete the whole app that they may not have the time to complete it all before they’re called back” (21, female, Black).

## Discussion

STI/HIV testing, and the prevention and reduction of drug use and sexual risk behaviors remain important strategies to prevent HIV among youth in the United States [40]. However, the scientific knowledge remains limited with respect to scalable mHealth solutions to improve uptake of STI and HIV testing and the prevention and reduction of drug use and sexual risk behaviors among youth [16]. Compared to AIDS service organizations and adult-focused clinics, youth are more likely to seek drug abuse and STI/HIV prevention and risk reduction services from youth-centered community health clinics [15]. Therefore, examining the feasibility and acceptability of the S4E preventive intervention has important public health implications, suggesting the potential high impact of S4E by targeting a culturally congruent setting that is frequently visited by vulnerable youth. Using a multi-methods approach, both quantitative and qualitative data suggest high feasibility and acceptability of S4E among youth and clinicians, indicating its potential as an mHealth solution to drug use and STI/HIV among vulnerable youth.

The findings support the notion that the feasibility of the S4E preventive intervention is high. Clinicians’ heavy caseload, coupled with time constraints, have led researchers and clinicians alike to question the feasibility of interventions in clinic settings [41]. However, the present study suggests that we successfully completed all study procedures, including the recruitment, engagement, and enrollment of a diverse sample of youth between the ages of 14–21, as well as clinician participants. We attribute this to our community–university approach, including the use of CBPR principles, which may enhance uptake of preventive interventions by the target community [17]. For example, the S4E intervention aligned with the clinic workflow, and achieved excellent completion rates of the S4E intervention and assessment procedures by both youth and clinician participants. Qualitative data from both youth and clinician individual interviews suggest the high feasibility of S4E.

Youth and clinician participants’ acceptability of the S4E mHealth preventive intervention was high. Among youth, participation in an intervention prior to their clinic visit was ideal: they expressed high satisfaction with both the youth-facing S4E app and experiences related to the youth-clinician prevention and risk reduction encounter, as well as high willingness to recommend S4E to friends, suggesting high acceptability of S4E. In fact, the findings highlight how S4E prepared youth to discuss drug use and sexual risk behaviors, reduced their feelings of discomfort related to engaging with clinicians in risk communication, facilitated targeted and tailored drug abuse and STI/HIV prevention strategies, and assisted with linkage to prevention and care services, including STI and HIV testing. Among clinicians, high satisfaction with the user interface and user experience of the clinician-facing S4E app, interest in using S4E for future appointments, and meaningful experiences with regard to the youth-clinician encounter, as well as high willingness to recommend S4E to colleagues, underscore clinicians’ high acceptability of S4E. Specifically, our data suggest that the S4E approach optimally identified for clinicians the most salient risk behaviors endorsed by youth, prepared clinicians and their patients to engage in risk communication, and facilitated targeted and tailored drug abuse and STI/HIV prevention and risk reduction strategies. The present findings support prior research suggesting youths’ high usability and acceptability of the youth-facing S4E app [25] and expands on this research by examining whether and to what extent including a clinician-facing app to facilitate a youth–clinician encounter aimed at reinforcing prevention and risk reduction messaging is acceptable to both youth and clinicians.

We explored uptake of STI and HIV testing immediately post-intervention. Notably, all youth who reported past 90-day condomless vaginal or anal sex—prominent sexual risk behaviors that enhance vulnerability of STI and HIV infection [6]—or having never been tested for STIs or HIV in their lifetime, were tested for both STIs and HIV immediately post-intervention. Consequently, the present findings suggest that the intervention may have an impact on screening, testing, and linking vulnerable youth to prevention and care services. In fact, both youth and clinicians shared their experiences related to how the S4E intervention prepared them to discuss STI and HIV testing, which, in turn, may have enhanced uptake of STI and HIV testing among youth. In addition, both youth and clinicians described addressing drug use and sexual risk behaviors during the clinic visit, an event that some youths had not experienced in prior clinic visits. This is especially noteworthy given that assessing youths’ drug use and sexual risk behaviors in clinic settings and linking them to prevention and care services are federal priorities [40]. Given our design, however, we cannot determine whether uptake of STI and HIV testing, as well as drug use and sexual risk behaviors communication, is a result of community practice or the S4E preventive intervention. Future research should examine the effects of the S4E intervention on youths’ uptake of STI and HIV testing and prevention and reduction of drug use and sexual risk behaviors in a randomized controlled trial design.

The present study has important theoretical implications. Indeed, the prevention principles affirm the need for preventive interventions to be theory-driven, as well as for them to take a multilevel approach to the prevention of drug abuse and STI/HIV among youth [18]. Guided by empowerment [20] and ecodevelopmental [21] theories, the S4E intervention takes a multilevel approach by targeting both intrapersonal (i.e., drug use and STI/HIV knowledge, self-efficacy, refusal skills) and interpersonal (i.e., clinician–youth risk communication) levels that shape youth drug use and sexual risk behaviors. Qualitative data from both youth and clinicians suggest that the S4E intervention may improve youths’ drug use and STI/HIV knowledge, self-efficacy, and refusal skills, as well as enhance youth–clinician risk communication. The findings lend support that youth drug use and STI/HIV knowledge, self-efficacy, and refusal skills, as well as youth–clinician risk communication are potential pathways by which changes in youth risk behaviors occur. Furthermore, the findings suggest that integrating empowerment [20] and ecodevelopmental [21] theories may provide a robust framework to guide the S4E preventive intervention. However, our design in the present study does not allow us to formally test S4E’s potential mechanisms of change. Few researchers have determined the pathways through which youth behavior changes occur, especially regarding technology-based interventions [16]. Therefore, future research examining the utility of this framework in an experimental design to test the hypothesized mechanisms of change is warranted.

The present study has several limitations that should be mentioned. First, we caution that our findings were drawn from a small sample that is not representative of the United States youth population, and so the findings may not generalize to all youth. However, our sample size aligns with research focused on examining the feasibility and acceptability of technology-based HIV interventions where sample sizes have ranged from 10–31 [34–37]. A second limitation is that the intervention was delivered in only one youth-centered community health clinic. While the findings may not be generalizable, our results provide some evidence of clinicians’ high acceptability and feasibility of delivering a multilevel mHealth preventive intervention in a clinic setting. A third limitation is the lack of access to the youths’ medical records and the reliance on self-reported measures. This approach may lead to youths under-reporting drug use and sexual risk behaviors [42]. Although researchers suggest that self-report measures of risk behaviors may be reliable [43], future research could benefit from collecting biomarker data in combination with self-report data [44]. Research on the collection of biomarker data among youth is mixed. Whereas some researchers report that the collection of biomarker data among youth is acceptable [45–46], others suggest that youth perceive the collection of these data as punitive and signals the research team’s mistrust toward the youth [47]. Therefore, more research, especially in clinic settings, is needed. Finally, our one-arm pilot study design is a limitation. Although this approach aligns with published pilot studies focused on determining the feasibility and acceptability of preventive interventions [48], a two-arm design can provide data on the relative acceptability of a technology-based intervention [49].

Advancements in prevention science and technology science in conjunction with the ubiquity of mobile devices among youths provide prevention researchers with innovative tools to develop and test scalable mHealth solutions to drug use and STI/HIV. Although technology-based [50] and clinic-based [51] interventions have been used in isolation in prior drug abuse and STI/HIV prevention research, efforts to understand how technology can be leveraged in youth-centered community health clinic settings—an ideal setting evidenced by youths’ frequent visits—in combination with clinician engagement to reinforce prevention and risk reduction strategies is warranted. The results from the present study suggest both youths’ and clinicians’ high acceptance of the S4E preventive intervention and high feasibility in the context of the clinic workflow. Determining the feasibility and acceptability of the S4E intervention contributes to the scientific knowledge of technology-based interventions and is an important step in working toward establishing S4E as a scalable mHealth solution to drug abuse and STI/HIV among vulnerable youth.
